# A new species of *Grotea* Cresson (Hymenoptera, Ichneumonidae, Labeninae) from Colombia

**DOI:** 10.3897/zookeys.389.6066

**Published:** 2014-03-14

**Authors:** Andrés Fabián Herrera-Flórez

**Affiliations:** 1 University of Manitoba, Department of Entomology, 214 Animal Science Bldg, Winnipeg, Manitoba, Canada R3T 2N2

**Keywords:** Ichneumonoidea, Groteini, South America, Neotropics, taxonomy, bee parasitoid, *Chilicola*, Labeniformes, host record

## Abstract

The genus *Grotea* has 18 described species. A new species, *Grotea villosissima* **sp. n.**, is described here and its host information included. This is the first record of *Grotea* for Colombia.

## Introduction

The Labeninae is a group of parasitoid wasps with a mainly Gondwanan distribution (Townes 1969; Gauld 1983; Wahl 1993, Gauld 2000); although most of its members occur in the Australasian or the Neotropical region, two of its genera, *Labena* and *Grotea*, occur in North America (Townes and Townes 1960; Slobodchikoff 1970, Gauld 2000). According to Gauld and Wahl (2000), those exceptions provide evidence of the spread of some members of this subfamily towards the north after the establishment of the Mesoamerican land bridge (Gauld and Wahl 2000, Gauld 2000).

The described species of *Grotea* can be grouped in 4 species-groups: *Grotea anguina* species-group, *Grotea superba* species-group, *Grotea chiloe* species-group and *Grotea gayi* species-group (Wahl 1993). According to Gauld and Wahl (2000) *Grotea* originated in southern South America, diversified in tropical South America, and colonized North America recently across the Mesoamerican land bridge. Proof of this is that the more basal taxa of *Grotea* are endemic to Chile and that the richness of species and the richness of species groups are highest in South America. *Grotea* comprises 18 species (Yu et al. 2005): 9 occur in South America, 8 in Central America and 3 in North America (Yu et al. 2005). From the 4 species-groups of *Grotea*, the *anguina* species-group is the only one that occurs outside the Gondwanan region (South America, south of equator) (Gauld 2000).

*Grotea* species parasitize bee hosts of the genera *Ceratina* Latreille, 1802 (Graenicher 1905; Rau 1928; Daly et al. 1967; Slobochikoff 1970), *Chilicola* Spinola, 1851 (Packer 2004; González and Giraldo 2009), *Megachile* Latreille, 1802 and *Manuelia* Vachal, 1905 (Janvier 1967; Gauld 2000; Gauld and Wahl 2000).

The aim of this paper is to describe a new species collected from a nest of *Chilicola* (subgenus *Oroediscelis*) *deborahae* Gonzalez, 2009 (Gonzalez and Giraldo 2009) found in Boyaca, Colombia. This is the first record of *Grotea* for Colombia.

## Material and methods

A nest of *Chilicola* (Colletidae) was found by my colleague Victor González in dry branches of *Espeletia argentea*. A *Grotea* specimen emerged from one of the cells. After comparing this specimen with the descriptions made by Cameron (1886), Cresson (1864, 1874, 1879), Gauld (2000), Porter (1989), Schmiedeknecht (1907), Slobodchikoff (1970), Spinola (1851), Thunberg (1822) and Townes and Townes (1960) it was clear that the specimen belongs to a new species. The morphological terminology used in the description of *Grotea villosissima* sp. n. follows Gauld (1991, 2000).

The holotype is preserved at the Museo de Entomología “Francisco Luis Gallego”, Universidad Nacional, sede Medellín (UNCM).

## Systematics

### Genus *Grotea* Cresson, 1864

#### Grotea villosissima

Herrera-Flórez sp. n.

http://zoobank.org/C7B7AD5D-68C4-49BF-84E1-139B6EA04070

http://species-id.net/wiki/Grotea_villosissima

Figures 1 –10

##### Material examined.

*Holotype*: female, “COLOMBIA: Boyacá: Arcabuco. Santuario de Fauna y Flora de Iguaque, Camino de la Laguna, 5°70'N, 73°46'W, 3400–3600m, emergió de celda en nido de *Chilicola (Oroediscelis)* sp. n. (Apoidea, Colletidae). Ese nido estaba en ramas secas de *Espeletia argentea*, 23 Agosto 2003, leg.V.González (UNCM)”.

##### Diagnosis.

**Figures 1–5.**
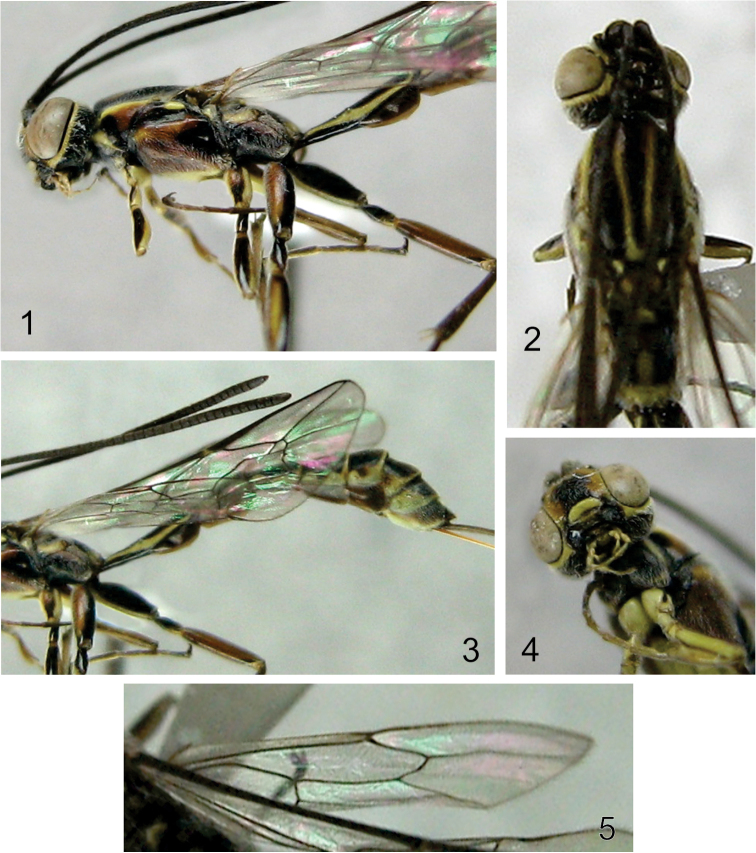
Photographs of *Grotea villosissima* sp. n. **1** Head, mesosoma and first tergite, lateral view **2** Head, mesosoma, dorsal view **3** Part of mesosoma and metasoma, lateral view **4** Head and part of mesosoma, ventral view **5** Hind wing.

**Figures 6–10.**
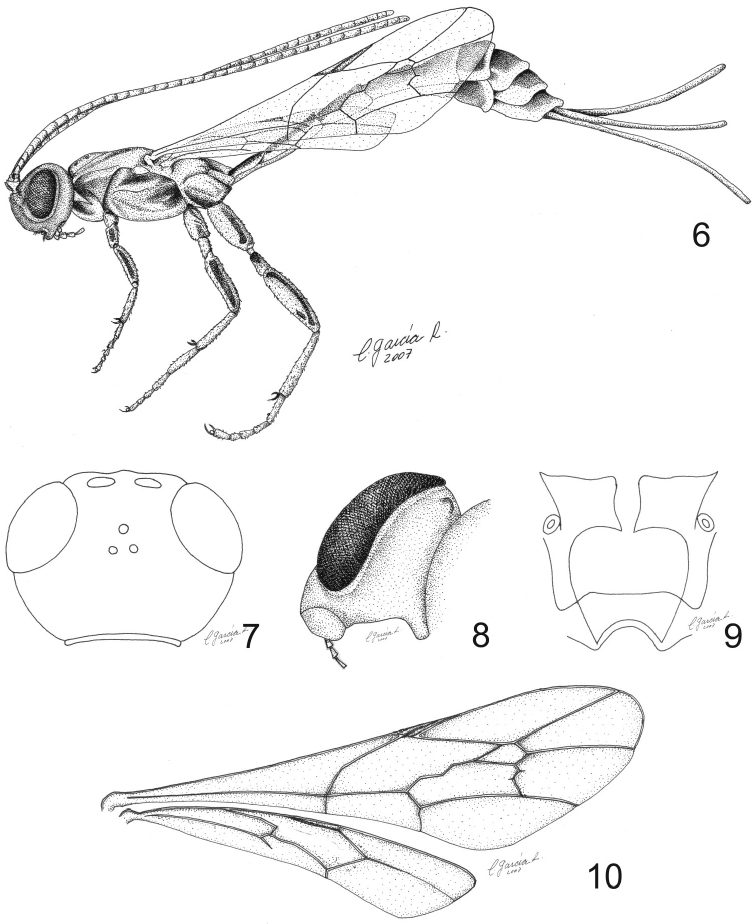
Line drawings of *Grotea villosissima* sp. n. **6** Habitus **7** Head, posterodorsal view **8** Head (showing detail of genal projection), posteroventral view **9** Propodeum, dorsal view **10** Wings.

This new species can be recognized from other described species of *Grotea* by the following combination of characters: gena close to the junction of occipital and hypostomal carinae with inwards genal projections (Fig. 8); propodeum with anterior transverse carina centrally weak and indented (Fig. 9); metasoma with tergite I slender but slightly shorter than mesosoma and rather straight (Figs 1, 3, 6); ovipositor shorter than the fore wing (Fig. 6).

##### Description.

Female. Fore wing 7.4 mm long.

Head in dorsal view with gena behind eyes rounded (Fig. 7); posterior ocellus separated from eye by 1.7 × its own maximum diameter (Fig. 7); genal projection present, laterally indistinct, ventroposteriorly evident; projection narrowing apically, horizontally oriented to inside of oral cavity; genal projections almost touching each other (Fig. 8); antenna with 35 flagellomeres (Fig. 6); flagellomere I 1.0 × as long as flagellomeres II and III combined. Epomia absent.

**Mesosoma.** Mesoscutum smooth with isolated inconspicuous punctures; scutellum in profile weakly convex; hind wing with *Cu*1 strongly pigmented, not reaching margin (Figs 5, 10); propodeum (Fig. 9) 2.1–2.2 × as long as broad; anterior transverse carina complete, centrally weak and indented, thus not forming a smooth arc from side to side (area basalis posteriorly enclosed); pleural carina complete; posterior transverse carina complete, although laterally weak; lateral longitudinal carina complete (area spiracularis enclosed); lateromedian longitudinal carina reaching anterior transverse carina, then absent; area lateralis not enclosed internally, rectangular, about 2.3 × as long as broad, with posterolateral corner at right angle removed from lobe surrounding coxal insertion. Area superomedia not differentiated, basally and distally weak, laterally open.

**Metasoma.** Tergite I (Figs 1, 3, 6) straight (not bowed upwards) and slender, shorter than mesosoma (mesosoma 1.5 × as long as tergite I); tergite I at least 4 times as long as broad posteriorly; visible part of ovipositor 2.7–2.9 × as long as hind tibia (Fig. 6).

**Color.** (Figs 1–5).

A predominantly black species with head with yellow circumocular area and clypeus. Pronotum with two longitudinal yellow spots, along ventral and dorsal margins, and a submedial red spot towards posterior margin. Mesopleuron mostly red with two large black areas, one at epicnemium and other towards posterior margin, and a yellow spot close to tegula. Mesoscutum with yellow longitudinal spots distally. Scutellum mostly brownish anteriorly. Propodeum with brownish area basalis, area superomedia with yellow central spot, yellow areae petiolaris, posteroexterna and spiracularis, dull yellow area lateralis. Metasoma with extensively yellow marked tergites. Dull yellow ovipositor sheath. Fore and mid legs with extensively yellow-marked coxae and femora. Hind leg with extensively red marked coxa and femur. Fore wing hyaline.

**Pubescense.** Gena, vertex, mesosoma and metasoma with dense, whitish setae, setae longer on propleuron, pronotum, mesopleuron and metapleuron.

##### Etymology.

The name of this new *Grotea* species refers to its uncommon pubescence.

##### Discussion.

The 12 described species of the *Grotea anguina* species-group have backwards-directed genal projections whilst *Grotea villosissima* sp. n. has inwards-projecting genal projections (Fig. 8).

The three described species of the *Grotea chiloe* species-group have an upwards bowed first tergite, whilst *Grotea villosissima* sp. n. has a straight first tergite (Figs 1, 3, 6).

The two described species of the *Grotea gayi* species-group lack genal projections, have a strong epomia and have a fully closed area superomedia. *Grotea villosissima* sp. n. has genal projections (Fig. 8), lacks epomia and has an incomplete area superomedia (Fig. 9).

*Grotea superba*, the only described species of the *Grotea superba* species-group, is similar to *Grotea villosissima* sp. n. in having a straight first tergite (Figs 1, 3, 6). However, *Grotea superba* has sharp downwards-projecting genal projections, 43 flagellomeres, 9.0–12.5 mm fore wing length, hind wing with a strongly pigmented *Cu*1 reaching the margin of the wing, and ovipositor always longer than the fore wing. *Grotea villosissima* sp. n. has inwards-projecting genal projections, 35 flagellomeres, 7.4 mm fore wing length, hind wing with *Cu*1 not reaching margin of the wing (Fig. 5, 10) and ovipositor shorter than the fore wing (Fig. 6). Finally, there are also clear differences in the color pattern between these two species (e.g. black flagellum with a white ring between flagellomeres 24 to 42 in *Grotea superba* and a black flagellum (Figs 1, 3) in *Grotea villosissima* sp. n.). All the differences between *Grotea villosissima* sp. n. and the described species of *Grotea* make the inclusion of this new species into any of the species-groups proposed by Wahl (1993) uncertain.

*Grotea villosissima* sp. n. is the first species of *Grotea* recorded from Colombia, where at least 5 more species of this genus occur (Gonzalez and Giraldo 2009).

## Supplementary Material

XML Treatment for Grotea villosissima
